# Characterisation of molecular events following cisplatin treatment of two curable ovarian cancer models: contrasting role for p53 induction and apoptosis *in vivo*

**DOI:** 10.1038/sj.bjc.6602167

**Published:** 2004-09-28

**Authors:** P A Clarke, K E Pestell, F Di Stefano, P Workman, M I Walton

**Affiliations:** 1 Cancer Research UK Centre for Cancer Therapeutics, Institute of Cancer Research, Haddow Laboratories, Sutton, Surrey SM2 5NG, UK

**Keywords:** chemotherapy, cisplatin, microarray, ovarian cancer, p53, apoptosis

## Abstract

The detailed molecular basis and determinants of *in vivo* tumour sensitivity to conventional anticancer agents remain unclear. We examined the cellular and molecular consequences of cisplatin treatment using two ovarian tumour xenograft models that had not been previously adapted to culture *in vitro*. Both xenografts were curable with clinically relevant multiple doses of cisplatin. Following a single dose of cisplatin (6 mg kg^−1^ i.p.) growth delays of 25 and 75 days were obtained for pxn100 and pxn65, respectively. This difference in response was not due to differences in DNA damage. Pxn100 tumours had a functional p53 response and a wild-type p53 sequence, whereas pxn65 harboured a mutant p53 and lacked a functional p53 response. Microarray analysis revealed the induction of p53-regulated genes and regulators of checkpoint control and apoptosis in pxn100 tumours following cisplatin-treatment. By contrast, there was no p53-dependent response and only limited changes in gene expression were detected in the pxn65 tumours. TUNEL analysis demonstrated high levels of apoptosis in the pxn100 tumours following cisplatin treatment, but there was no detectable apoptosis in the pxn65 tumours. Our observations show that a marked *in vivo* response to cisplatin can occur via p53-dependent apoptosis or independently of p53 status in human ovarian xenografts.

*cis*-Diamminedichloroplatinum(II) or cisplatin (CDDP) is an important anticancer agent widely used to treat many malignancies and is the single most effective chemotherapeutic agent used in the treatment of ovarian cancer (reviewed by Chu, 1994; Adams *et al*, 1998). The major mechanism of CDDP cytotoxicity is considered to result from DNA damage caused by the formation of DNA adducts, including inter- and intrastrand crosslinks. The principle mechanisms that translate CDDP-induced DNA damage into cytotoxicity are not understood, although there is considerable evidence that CDDP treatment can induce a cell cycle arrest and/or apoptosis (Eastman, 1999).

Several factors are reported to influence sensitivity or response to CDDP-treatment, including intracellular drug uptake, DNA binding and repair as well as thiol binding (reviewed in Kartalou and Essigmann, 2001) However, the role of the p53 tumour suppressor gene has been of particular interest in attempts to understand the cellular response to anticancer agents such as CDDP, as it has been demonstrated that functional p53 is required for induction of apoptosis in lymphoid tumours and transformed primary cell line models following genotoxic damage induced by cytotoxic drugs or irradiation (Lowe *et al*, 1993; Komarova *et al*, 1997). p53 is the most frequently mutated tumour suppressor in human malignancy and mutations have been detected in 40–80% of ovarian cancers (Reles *et al*, 2001). However, its role in determining sensitivity of ovarian cancer cells to CDDP remains unclear as the literature shows that the presence or absence of p53 can cause resistance, sensitivity or have no effect (Brown *et al*, 1993; Eliopoulos *et al*, 1995; Herod *et al*, 1996; Wu and El-Deiry, 1996; De Freudis *et al*, 1997; O'Connor *et al*, 1997; Pestell *et al*, 1998; 2000).

Another factor that influences response to CDDP is the capacity to undergo apoptosis. Treatment of ovarian cancer cells results in the induction of apoptosis following a robust activation of Jun N-terminal protein kinases by c-ABL, a response not seen in resistant cells (Hayakawa *et al*, 1999; Gebauer *et al*, 2000). Activation of c-ABL by CDDP requires the ATM protein, DNA-PK and a functional DNA mis-match repair response (Kharbanda *et al*, 1998). Expression of the BCL-2 family of pro- and anti-apoptotic genes also affects the apoptotic response following CDDP treatment; however, again in human ovarian cancer the evidence is contradictory (Eliopoulos *et al*, 1995; Herod *et al*, 1996; Perog *et al*, 1996; Wu and El-Deiry, 1996; Jones *et al*, 1998; Beale *et al*, 2000).

The translation of molecular mechanisms of action of anticancer agents from *in vitro* tissue culture models to tumours *in vivo* can be challenging. *In vitro* tissue culture models are extremely useful for examining the response of ovarian cancer cells to CDDP and play a role in dissecting molecular events following exposure to CDDP. Comparison of ovarian tumours treated *in vivo* and *in vitro* with CDDP have demonstrated that their relative sensitivity observed *in vitro* is preserved *in vivo* (Kelland *et al*, 1992). However, use of tumour cells that have been adapted to growth on a plastic surface may have limitations as it is frequently necessary to employ concentrations considerably higher than those achieved in patients to detect measurable effects *in vitro* (eg Vermorken *et al*, 1984; Potapova *et al*, 1997). Moreover, *in vitro* approaches to understand factors that influence response to anticancer agents commonly generate degrees of resistance factors that are higher than that observed in patients (De Graeff *et al*, 1988; Teicher *et al*, 1991). Several studies have also noted that CDDP-resistant tumours established by *in vivo* treatment with CDDP show acquired resistance in three-dimensional spheroid *in vitro* cultures, but not when cultured as monolayers *in vitro* (Teicher *et al*, 1990; Kobayashi *et al*, 1993).

In this study we set out to explore the molecular events following a robust response to CDDP *in vivo*, using two sensitive human ovarian cancer xenograft models. These xenografts were selected deliberately because they were curable by multiple clinically relevant doses of CDDP, had not been previously adapted to *in vitro* culture conditions and could not be passaged *in vitro*. We describe the measurement of molecular events following exposure to a single treatment with CDDP and demonstrate that a marked therapeutic response to CDDP occurs in the p53 wild-type tumour with evidence of apoptosis. Moreover, we also demonstrate an even greater response in a tumour that harbours mutant p53 and does not exhibit detectable apoptosis. Hence, *in vivo* sensitivity of ovarian cancers to CDDP can occur in the presence or absence of both a p53-dependent response and also of apoptosis.

## MATERIALS AND METHODS

### Tumour xenografts

All animal studies were carried out in accordance with UKCCCR guidelines and after independent ethical committee approval (Workman *et al*, 1998). Animals were housed in Maxi-miser PIV (positive individual ventilated) caging systems (Thoren, Hazleton, Pennsylvania, USA) and maintained on a Labsure 21% protein diet with access to tap water *ad libitum*. The pxn65 xenograft was derived from a moderately differentiated adenocarcinoma and was epithelial in origin. By contrast, the pxn100 xenograft was derived from an endodermal sinus tumour and was germ cell in origin. Their xenograft histologies reflected those of the tumour of origin (Harrap *et al*, 1990). The pxn65 and pxn100 human ovarian cancer xenografts were passsaged as or grown for treatment from 2 mm^3^ biopsy fragments implanted s.c. in anaesthetised female nude (random bred) mice (age 6–8 weeks). Mice bearing comparable sized tumours (approx 8 mm^3^ diameter) were randomised into treatment groups or control groups. Cisplatin (Sigma-Aldrich, Gillingham, Dorset, UK) was dissolved in 0.9% saline immediately prior to use. For antitumour studies treatment was a single i.p. dose of 6 mg kg^−1^ CDDP and tumour diameters were measured every 7 days. For other studies, where indicated, mice were irradiated with 5 Gy from a ^60^Co source. Tumours were rapidly removed from mice and a portion immediately snap frozen on dry ice or homogenised in lysis buffer, prior to analysis.

### Measurement of CDDP-induced DNA adducts by quantitative-PCR

Genomic DNA was extracted from tumour samples by incubation for 2 h at 56°C in extraction buffer (100 mM NaCl, 50 mM Tris HCl pH 7.6, 1% SDS, 50 mM EDTA pH 8.0, 100*μ*g ml^−1^ proteinase K). DNA was recovered by the addition of an equal volume of 5 M LiCl and two volumes of chloroform. The aqueous phase was recovered and DNA was precipitated by the addition of two volumes of absolute ethanol and quantified spectrophotometrically at 260 nm. Quantification and integrity of genomic DNA was confirmed by electrophoresis of 1 *μ*g on a 0.6% agarose gel. A quantitative-PCR approach was used to measure DNA adducts on the genomic DNA of the treated tumours as Taq DNA polymerase cannot progress past certain DNA-CDDP adducts (Grimaldi *et al*, 1994). The assay employed two primers (NRAS1-5′-CCT AAA TCT GTC CAA AGC AGA GGC-3′, NRAS2-5′-CAG CAA GAA CCT GTT GGA AAC CAG-3′) that yielded a 523 bp of the first intron/exon of *N-RAS*. Calibration experiments determined that 25 cycles of 1 min at 94°C, 1 min at 65°C and 2 min at 72°C were optimal for quantification (data not shown). Briefly, 0.5 *μ*g of genomic DNA was amplified with 1 *μ*M primers, one of which, NRAS1, had been end-labelled by incubation with polynucleotide kinase and [*γ*^32^P]ATP (Clarke, 1999). Products were separated on a denaturing 6% polyacrylamide gel containing 8 M urea, vacuum dried onto Whatman No. 1 paper and the product quantified by phosphorimaging using a Molecular Dynamics Storm phosphorimager (Amersham Biosciences UK Ltd, Little Chalfont, Buckinghamshire, UK).

### Immunoblotting

Xenografts were removed and homogenised in lysis buffer (150 mM NaCl, 50 mM Tris, 1% NP40, 0.2% SDS, 1 mM PMSF, 10 *μ*g ml^−1^ aprotinin, 10 *μ*g ml^−1^ leupeptin and 1 mM sodium orthovanadate). Protein concentration was determined using a BCA protein assay (Pierce Biotechnology Inc., Rockford, IL, USA). Samples (100 *μ*g) and Rainbow™ molecular weight markers (Amersham Biosciences UK Ltd, Little Chalfont, Buckinghamshire, UK) were separated by electrophoresis on polyacrylamide gels and electro-transferred onto Hybond-c nitrocellulose membranes (Amersham Biosciences UK Ltd, Little Chalfont, Buckinghamshire, UK). To avoid potential cross-reaction with host stroma and infiltrating host lymphocytes, immunoblots were probed with non-rodent antibodies. Immunoblots were blocked with 5% non-fat milk in TBST (10 mM Tris HCl pH 7.6, 142 mM NaCl, 0.1% Tween-20) and then incubated with a 1 : 5000 dilution of anti-Rho-GDI/D4 rabbit polyclonal antibody that recognises the full length and cleaved protein, 1 : 1000 dilution of an anti-procaspase 3 rabbit polyclonal or 1 : 1000 dilution of anti-bax rabbit polyclonal antibody (BD Biosciences Pharmingen, San Diego, CA, USA). Specific antigen-antibody interaction was detected with a horseradish peroxidase-conjugated anti-rabbit IgG using enhanced chemiluminscence Western blotting detection reagents (Amersham Biosciences UK Ltd, Little Chalfont, Buckinghamshire, UK).

### RNA analysis

Xenografts were removed and homogenised in 4 M guanidine thiocynate, 3 M sodium acetate pH 6.0, and 0.12 M *β*-mercaptoethanol. Total RNA was recovered by centrifugation through a 5.7 M caesium chloride gradient followed by ethanol precipitation. Total RNA (20 *μ*g) was separated on a 1.2% agarose gel in the presence of formaldehyde and transferred by capillary action onto nylon Hybond-N membrane (Amersham Biosciences UK Ltd, Little Chalfont, Buckinghamshire, UK). RNA was fixed by baking at 80°C for 2 h and UV crosslinking at 1200 J m^−2^. *P21*^*waf1/cip1*^ full-length cDNA (2.12 kb), *MDM2* (0.9 kb fragment) and rat *GAPDH* cDNA fragment (1.3 kb) were labelled by random priming in the presence of [*α*^32^P]dCTP (Amersham Biosciences UK Ltd, Little Chalfont, Buckinghamshire, UK). Probes were separated from unincorporated dNTPs using Microspin™ S-300 HR columns (Amersham Biosciences UK Ltd, Little Chalfont, Buckinghamshire, UK), hybridised using the method of Church and Gilbert (1984) and the signal detected by direct autoradiography.

Single-stranded, high-specific activity antisense RNA probes for human *Caspase 8*, *Fas-ligand*, *Fas-receptor*, *FADD*, *BCL-W*, *BCL-X*, *BFL-1*, *BID*, *BIK*, *BAK*, *BAX*, *BCL-2*, *MCL-1*, *RPL32* and *GAPDH* (BD Biosciences Pharmingen, San Diego, CA, USA) were prepared by *in vitro* transcription in the presence of [*α*^32^P]-UTP (Clarke, 1999). RNase protection was performed on 10 *μ*g of total RNA as described previously (Ross *et al*, 2001). Protected fragments were separated by electrophoresis on denaturing 5% polyacrylamide/7M urea gels, the gels were transferred to Whatmann No. 1 paper and vacuum dried. Protected probe fragments were detected and quantified by phosphorimaging using a Molecular Dynamics Storm phosphorimager and ImageQuant software (Amersham Biosciences UK Ltd, Little Chalfont, Buckinghamshire, UK).

The expression levels of 256 genes whose products are involved in the regulation of cell death and the cell cycle were profiled using a commercially available nylon microarray (BD Biosciences Clontech, Palo Alto, CA, USA). Poly(A)^+^ mRNA was prepared from total RNA using oligo(dT) cellulose (Invitrogen, Carlsbad, CA, USA). In total, 1 *μ*g of Poly(A)^+^ mRNA was labelled for 25 min at 50°C by reverse transcription with MMLV reverse transcriptase from gene-specific primers in the presence of [*α*^32^P]dATP (Amersham Pharmacia Biotech UK Ltd, Little Chalfont, Buckinghamshire, UK). Unincorporated nucleotides were removed using Chromaspin-200 columns (BD Biosciences Clontech, Palo Alto, CA, USA). The labelled probes were denatured for 20 min at 68°C in 0.1 mM NaOH, 1 mM EDTA, neutralised by the addition of an equal volume of 0.5 M NaH_2_PO_4_ pH 7.0, 1 *μ*g ml^−1^ Cot-1 DNA for a further 10 min at 68°C and added to microarrays prehybridised for 30 min at 68°C with 5 ml ExpressHyb containing 0.1 mg ml^−1^ sheared salmon testes DNA. Hybridisation was overnight at 68°C and membranes were washed to a final stringency of 0.1 × SSC, 0.5% SDS at 68°C. Hybridisation signals were detected and quantified by phosphorimaging (Molecular Dynamics Storm phosphorimager, Amersham Biosciences UK Ltd, Little Chalfont, Buckinghamshire, UK). Gene exposures were normalised using all of the seven housekeeping genes found on the membrane: Ubiquitin, (UBQ), M26880; 14-3-3-zeta (YWHAZ), M86400; hypoxanthine phosphoribosyltransferase 1 (HPRT1), V00530; GAPDH (GAPH), XO1677; Tubulin-alpha ubiquitous, (K-ALPHA-1), K00558; HLA-C4-alpha (HLAC) M11886; Beta-actin (ACTB), X00351; 23 kDa highly basic protein, X56932; Ribosomal protein S9 (RPS9) U14971 (trivial name; abbrevated name; Genebank Accession number). The median values of the 24 h treated samples were divided by the median of the 0 h controls and the ratios presented in Table 1

**Table 1:** Gene expression affected by >2.5-fold following 6 mg kg^−1^ i.p. CDDP

**Acc. no.**	**Gene name**	**pxn65**	**pxn100**	**Comments**
X56932	Ribosomal protein L13a (RPL13A)	**0.187**	1.741	Component of the 60S ribosomal subunit
K00558	Tubulin-alpha (TUBA1)	**0.257**	**2.261**	Ubiquitous tubulin isoform, member of a family of microtubule structural proteins
AF001954	Inhibitor of growth family, member 1 (ING1)	**0.258**	1.330	Required for optimal p53 function, can mediate growth arrest, senescence and apoptosis
AF017988	Secreted frizzled-related protein 5 (SFRP5)	**0.280**	**2.625**	Secreted apoptosis-related protein family member, may modulate of Wnt signalling
U14971	Ribosomal protein S9 (RPS9)	**0.327**	1.147	Component of the small 40S ribosomal subunit
AF017986	Secreted frizzled-related protein 2 (SFRP2)	**0.340**	0.109	Secreted apoptosis-related protein family member, may modulate of Wnt signalling
M14505	Cyclin-dependent kinase 4 (CDK4)	**0.362**	1.124	Required for cell cycle progression, phosphorylates and inactivates pRb
*M15796*	*Proliferating cell nuclear antigen (PCNA)*	**0.387**	**5.863**	*Processivity factor for DNA polymerases δ and ɛ. Role in DNA repair synthesis*
M62402	IGFBP6 insulin-like growth factor-binding protein 6	1.101	**28.76**	Binds and modulates insulin-like growth factor activity
*AF010310*	*Proline dehydrogenase 1 (PRODH1/PIG6)*	*1.069*	**9.289**	*Generates reactive oxygen species following p53-induction*
U22398	Cyclin-dependent kinase inhibitor 1C (CDKN1C/p57/Kip2)	0.513	**7.714**	Inhibits several G1 cyclin/Cdk complexes, overexpression arrests cells in G1
U90313	Glutathione transferase omega (GSTTLp28)	0.722	**6.236**	May bind glutathione, but has no transferase or peroxidase activity
*AF010314*	*Ectodermal-neural cortex (ENC1/PIG10)*	*0.992*	**5.616**	*Nuclear matrix-associated phosphoprotein, binds actin and interacts with active pRb*
*L22474*	*BCL2-associated X protein (BAX)*	*0.542*	**4.428**	*Pro-apoptotic bcl-family member, required for apoptosis induced by chemotherapeutics*
U10564	WEE1+homolog (WEE1)	0.676	**4.293**	Tyrosine kinase, negatively regulates entry into mitosis, phosphorylates cdc2/cyclin B
M11886	MHC, class I, C (HLA-C)	0.833	**4.190**	Role in the immune system, presents peptides derived from endoplasmic reticulum
M26880	Ubiquitin C (UBC)	0.504	**4.134**	Polyubiquitin protein precursor that marks cellular proteins for degradation
*AF010309*	*Quinone oxidoreductase homolog (PIG3)*	*0.898*	**3.611**	*Quinone oxidoreductase homologue, long-lived reporter of p53 induction*
M25753	Cyclin B1 (CCNB1)	0.671	**3.097**	G2M cyclin, binds cdc2 to form the maturation-promoting factor
M34065	Cell division cycle (CDC25C)	1.325	**2.913**	Protein tyrosine phosphatase; dephosphorylates cdc2/cyclin B and triggers mitosis
Y00285	IGF2R insulin-like growth factor 2 receptor	0.949	**2.822**	Mediates signal transduction
U77949	Cell division cycle 6 homolog (CDC6)	0.661	**2.692**	Regulates early steps of DNA replication, binds PCNA and ATM-dependent checkpoint
U23765	BCL2-antagonist/killer 1 (BAK1)	0.768	**2.643**	Pro-apoptotic BCL-family member, required for apoptosis induced by chemotherapeutics
U69611	Disintegrin and metalloproteinase domain 17 (ADAM17)	0.989	**2.526**	TNF-*α* converting enzyme, binds mitotic arrest deficient 2 protein
L31951	Mitogen-activated protein kinase 9 (MAPK9/JNK2)	0.967	**0.366**	Jun N-terminal kinase, binds p53 and increases its *t*_1/2_ in non-stressed cells
AF010312	LPS-induced TNF-alpha factor (PIG7)	1.445	**0.274**	LPS-induced TNF-alpha factor; functions as a transcription factor
U34819	Mitogen-activated protein kinase 10 (MAPK10/JNK3)	1.733	**0.222**	Jun N-terminal kinase activated by apoptosis signal related kinase 1
M35410	Insulin-like growth factor binding protein 2 (IGFBP2)	0.511	**0.155**	Binds and modulates insulin-like growth factor activity

Italic text indicates genes reported to be transcriptionally regulated by p53. Bold text highlights gene expression altered by >2.5-fold. The columns headed pxn65 and pxn100 are ratios of CDDP-treated gene expression relative to pretreatment control.

.

### TUNEL assay

The TUNEL method was used to detect apoptosis *in situ*. Terminal deoxyncleotidyltransferase binds 3′ hydroxy termini of DNA formed during apoptosis and will catalyse the addition of biotinylated-deoxynucleotides. Tumours were removed, fixed in methocarne, paraffin-embedded and 12.5 *μ*M thick sections cut. The sections were de-paraffinised in Histoclear, rehydrated in Tris-buffered saline, permeabilised by incubation with 20 *μ*g ml^−1^ proteinase K for 30 min at 37°C and endogenous peroxidases were inactivated by incubation in 3% H_2_O_2_ for 5 min. The slides were equilibrated for 30 min in TUNEL equilibration buffer (Calbiochem, San Diego, CA, USA) and then incubated at 37°C in a humidified chamber for 90 min with a TUNEL labelling mix containing terminal deoxyncleotidyltransferase. Slides were blocked for 10 min at room temperature and incubated with streptavadin-horse radish peroxidase conjugate for 30 min at room temperature. Slides were washed in Tris-buffered saline and incubated with DAB substrate for 15 min. Slides were counterstained with methyl green. HL-60 cells treated with actinomycin D *in vitro* were included as a positive control for apoptosis (data not shown).

## RESULTS

### Choice of human ovarian cancer model and response to CDDP

A previous study of 16 ovarian tumours established as *in vivo* xenograft tumours direct from tumour biopsy material had established their relative sensitivity to treatment with four platinum-based agents (Harrap *et al*, 1990). Two of the 16 tumours, pxn65 and pxn100, were found to be highly chemosensitive and curable following treatment with multiple doses of CDDP close to those commonly used in the clinic (Harrap *et al*, 1990 and unpublished). The majority of ovarian cancers are epithelial in origin and pxn65, derived from a moderately differentiated adenocarcinoma, belonged to this class. The biopsy was derived from a previously untreated patient who went on to show a complete remission with CDDP (Harrap *et al*, 1990). Pxn100 was derived from an endodermal sinus tumour and was germ cell in origin. This biopsy was taken from a patient who showed complete remission to a number of platinum-based combination therapies prior to biopsy (Harrap *et al*, 1990). These two lines would not grow as monolayer cultures *in vitro* and could only be passaged as *in vivo* xenografts where their response and histopathology as a xenograft closely paralleled that observed in the clinic (Harrap *et al*, 1990). Therefore, pxn65 and pxn100 provided a valuable opportunity to study cellular and molecular events associated with CDDP treatment in highly sensitive, curable, *in vivo* models of human ovarian cancer.

**Figure 1:**
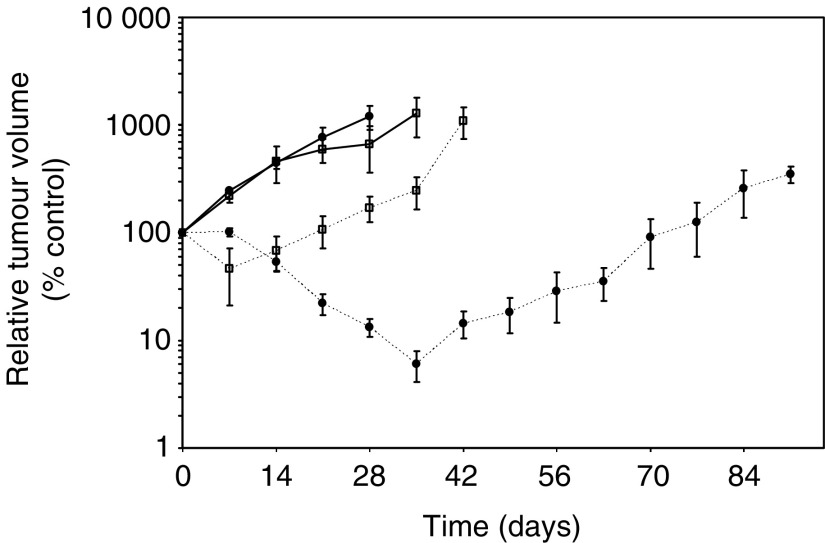
Growth curves of pxn65 and pxn100 xenograft tumour volume relative to pretreatment volume following a single treatment with CDDP (6 mg i.p. kg^−1^). Closed circular symbols=pxn65, open square symbols=pxn100, solid line=control, dotted line=CDDP; *n*=6, error bars=s.e.m.

Although both tumours responded to CDDP treatment, pxn100 was less sensitive as a greater number of treatments and a higher total dose (8 mg kg^−1^ i.p. on days 0, 7, 14 and 28) were required to achieve a cure in pxn100 xenografted mice compared to pxn65 (6 mg kg^−1^ i.p. on days 0, 49 and 77 Harrap *et al*, 1990 and unpublished). The different regimes required to achieve a cure would potentially complicate efforts to measure and compare responses following CDDP-treatment. Our aim here was to measure the early cellular and molecular events following CDDP treatment. Importantly, CDDP is rapidly sequestered by protein-binding, such that 60 min after administration no free drug remains in the plasma (Siddik *et al*, 1987). Therefore, with a single i.p. dose of 6 mg kg^−1^ we would be measuring response to a brief genotoxic insult induced by CDDP-treatment at a dose close to that administered in the clinic (the clinical dose is equivalent to approximately 2.5 mg kg^−1^). Both tumours initially responded to this treatment regimen. Pxn65 tumours did not show evidence for reduced relative tumour volume until day 14 (53.5%) and a nadir of 6% was seen at day 35 (Figure 1

). Pxn100 tumours showed evidence for reduced tumour volume 7 days following treatment with a minimum relative tumour volume of 46.4%. Pxn100 tumours exhibited a 25-day growth delay, whereas pxn65 tumours had a 75-day growth delay (Figure 1). This observation was surprising as the germ-line pxn100 tumour might be expected to be more sensitive to CDDP-treatment than the epithelial-derived pxn65 tumour (Ozols and Vermorken, 1997). The doubling times for the control tumours were similar at 5.0 and 5.8 days, respectively (Figure 1) and comparable to previous observations (Harrap *et al*, 1990). Therefore, differences in tumour growth rate were unlikely to account for their relative sensitivity to CDDP-treatment.

**Figure 2:**
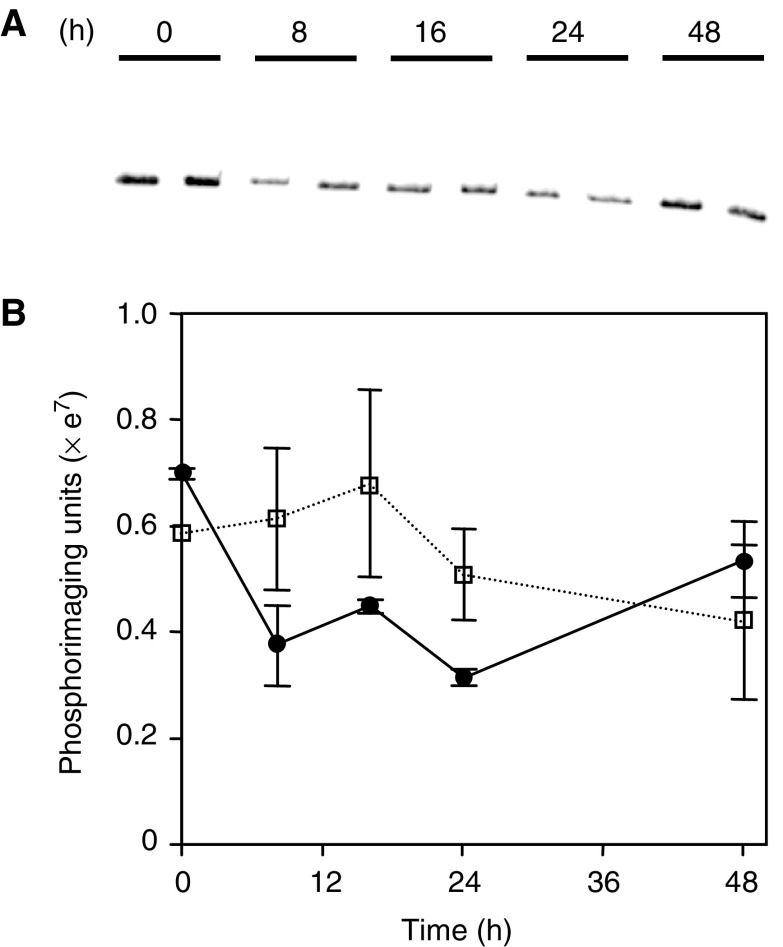
Quantitive PCR analysis of DNA adducts on genomic DNA from control and CDDP-treated (6 mg i.p. kg^−1^) pxn65 and pxn100 xenograft tumours. (**A**) Denaturing polyacryalmide gel analysis of the NRAS PCR product amplified from genomic DNA taken from duplicate pxn100 xenograft tumours. (**B**) Quantification of PCR products from analysis of CDDP-treated pxn65 and pxn100 tumours. Open square symbols, dotted line=pxn65 and closed circle symbols, solid line=pxn100 (*n*=2).

Differential sensitivity to single dose CDDP could be explained by differences in CDDP exposure, either due to reduced drug uptake or increased drug efflux. Alternatively, different DNA repair responses could influence the amount of DNA damage and response to this single dose of CDDP. Quantitation of the number of DNA adducts following treatment of the two tumours provides an estimate of the relative exposure to CDDP and the rate of DNA repair. To achieve this we used a quantitative-PCR approach to measure DNA adducts on the genomic DNA of the CDDP-treated tumours (Grimaldi *et al*, 1994; Koberle *et al*, 1996). Briefly, the quantitiative PCR-based assay involved amplifying a conserved 523 bp fragment of the *N-RAS* gene. DNA adducts will physically obstruct the polymerase and reduce product yield until the lesions have been removed or repaired. Decreased yields of the 523 bp PCR product, as a result of the formation of CDDP-adducts, were detected at 8 h (53% of pretreatment control) following CDDP treatment of pxn100 tumours (Figure 2

). The yield of PCR product was similar at 16 h (63% of control) and 24 h (44% of control), but had increased to 88% of the pretreated controls by 48 h suggesting that DNA damage repair processes were active. The number of DNA adducts detected in the pxn65 tumours was no greater than pxn100 tumours and at 48 h the yield of PCR product from CDDP-treated pxn65 (72% of control) and pxn100 tumours (88% of control) were similar. This implied that increased DNA-adduct formation and by extrapolation increased exposure to CDDP are unlikely to have contributed to the increased sensitivity of the pxn65 tumour. Although differences in inter- and intrastrand crosslinks as well as longer-term differences in DNA repair after 48 h could not be excluded by these data.

### P53 status of pxn65 and pxn100 tumours

**Figure 3:**
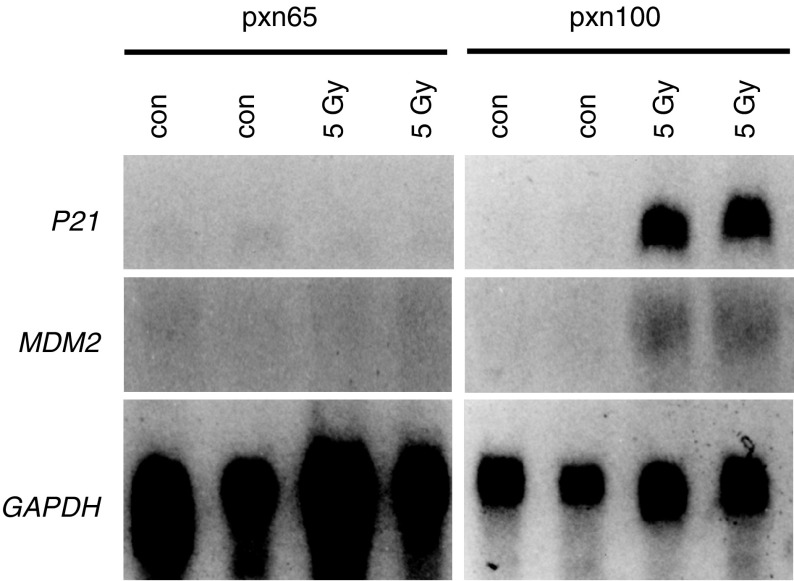
Northern blot analysis of *P21*^*waf1/cip1*^ and *MDM2* expression 4 h post-5 Gy irradiation of pxn65 and pxn100 xenograft tumours. Duplicate samples were analysed and *GAPDH* was included as a loading control.

p53 status has been reported to influence response to CDDP treatment in a number of models and consequently the p53 status of these two tumours were determined (Brown *et al*, 1993; Eliopoulos *et al*, 1995; Herod *et al*, 1996; O'Connor *et al*, 1997). In order to determine the functional p53 status in these two tumours we induced p53 by exposing pxn65 and pxn100 xenografts to ionising radiation and examined the expression of genes known to be increased following p53 induction. At 4 h after exposure to 5 Gy *γ*-irradiation *P21*^*waf1/cip1*^ and *MDM2* expression were increased in pxn100 tumours, but not pxn65 tumours (Figure 3

). This indicated that pxn100 tumours had a functional p53 response that was absent in pxn65 tumours. To confirm the p53 status of the tumours we used single-strand conformational polymorphism analysis and direct sequencing to determine that the pxn100 tumour had a wild-type p53 sequence and the pxn65 tumour had an insertion of GTGGTGAG and a deletion of base pair 868 in codon 290 (exon 8) of p53 (data not shown). This would result in a frame shift and premature termination at codon 306 that would yield an inactivate p53. Studies with a number of other DNA damaging anticancer drugs have demonstrated a clear correlation between p53 status and response (O'Connor *et al*, 1997). Previous *in vitro* studies have shown conflicting evidence for the role of p53 in CDDP sensitivity of ovarian cancer cell lines and it is possible that there is a subset of p53 mutant tumours that are CDDP sensitive. Nevertheless, our observation that the tumour with an inactivated p53 (pxn65) was more responsive to CDDP-treatment than the p53 wild-type (pxn100) tumour warranted further investigation of the molecular basis for this sensitivity.

### Analysis of apoptotic gene expression following CDDP treatment by RNase protection assay (RPA)

**Figure 4:**
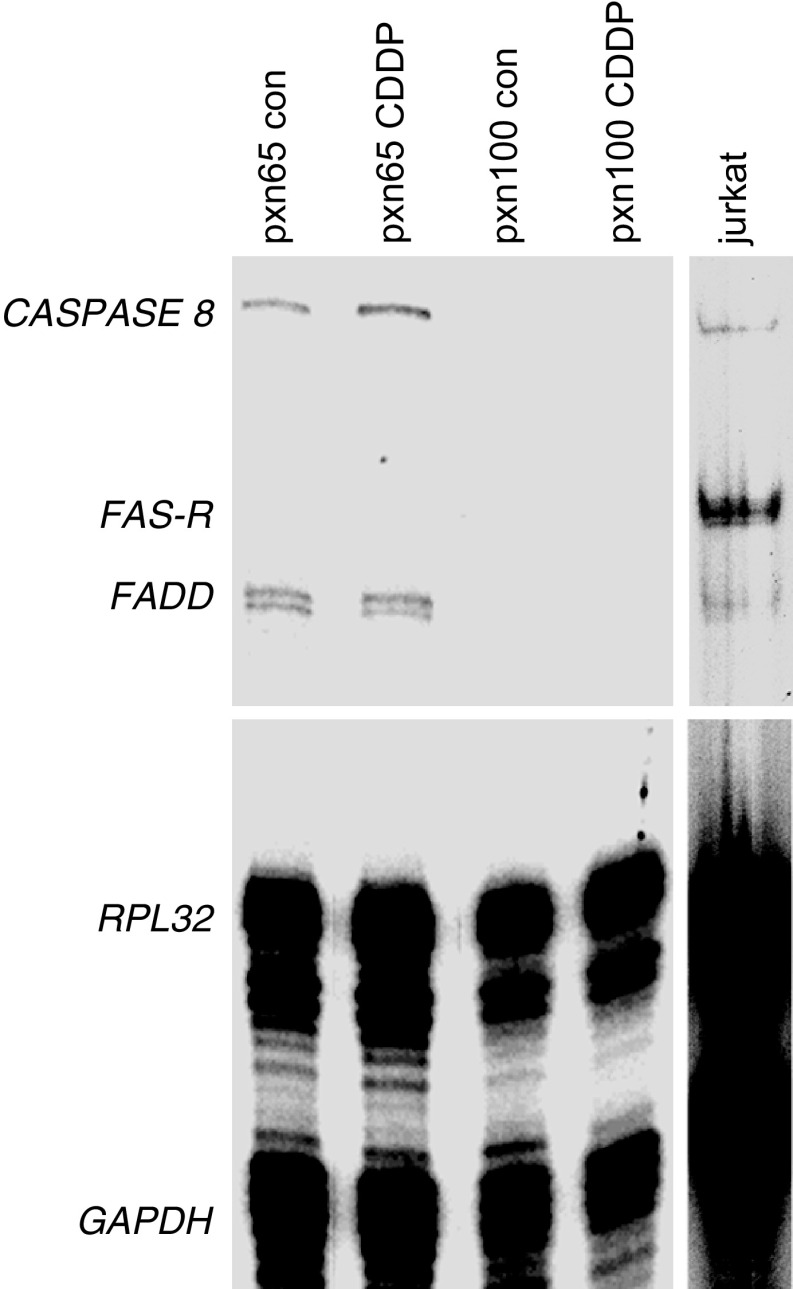
Analysis of *Caspase 8*, *Fas-ligand*, *Fas-receptor* and *FADD* expression by RNase protection analysis of RNA samples from pretreatment tumours and 24 h post-CDDP-treatment (6 mg kg^−1^ i.p.). Jurkat cells were included as a positive control for Fas-receptor expression. *RPL32* and *GAPDH* were included as loading controls.

**Figure 5:**
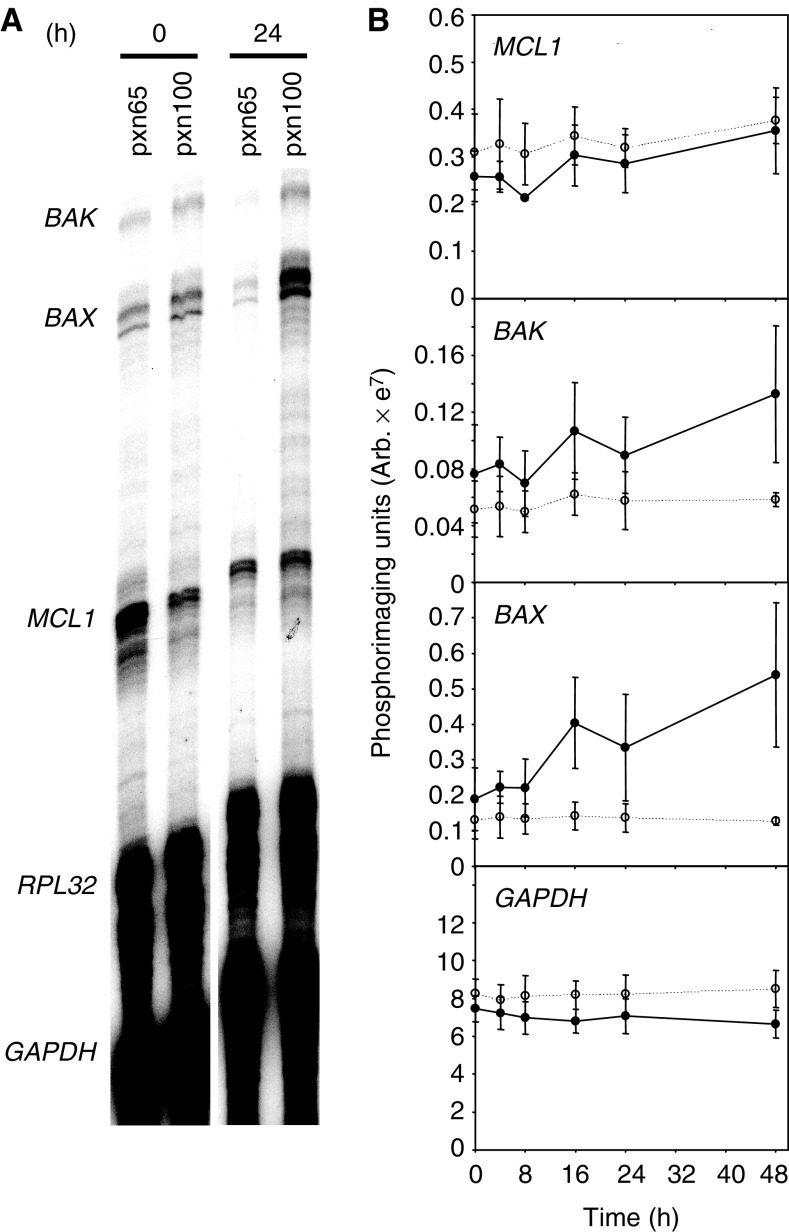
(**A**) Analysis of *BCL-2* family members by RNase protection analysis of RNA samples from pretreatment tumours and 24 h postcisplatin treatment (6 mg kg^−1^ i.p.). (**B**) Time course of CDDP-treatment (expression normalised to *RPL32*; open circle, dotted line=pxn65 tumours; filled circle, solid line=pxn100 tumours; *n*=3, error bars=s.e.m.).

One mechanism by which CDDP has been proposed to induce apoptosis is through increased expression of the Fas-receptor, a member of the tumour necrosis receptor superfamily, via activation of p53 (Muller *et al*, 1997; Fulda *et al*, 1998; Seki *et al*, 2000). RNase protection analysis demonstrated that pxn65 and pxn100 tumours lacked detectable expression of the *Fas-ligand* and *Fas-receptor* and there was no evidence for induction of their expression following CDDP treatment (Figure 4

). The expression of the BCL-2 family of genes that regulate apoptosis was also assayed. No measurable signal was detected for *BCL-W, BCL-X*, *BFL-1*, *BID*, *BIK* and *BCL-2*; however, similar levels of *MCL1*, *BAX* and *BAK* expression were detected in both tumours (Figure 5

). Following CDDP-treatment, pxn100 tumours exhibited evidence for increased *BAX* and *BAK* expression consistent with wild-type p53 function. This was time-dependent and reached a maximum of 2.9- and 1.7-fold, respectively, at 48 h. In contrast, CDDP-treatment of pxn65 tumours did not significantly alter *BAX* and *BAK* expression. Increased expression of BAX and BAK may be significant as the products of these genes have been reported to be involved in the induction of apoptosis following treatment with anticancer agents (Jones *et al*, 1998; Ionov *et al*, 2000; Zhang *et al*, 2000).

### cDNA microarray analysis of cell cycle and apoptosis gene expression following CDDP treatment

**Figure 6:**
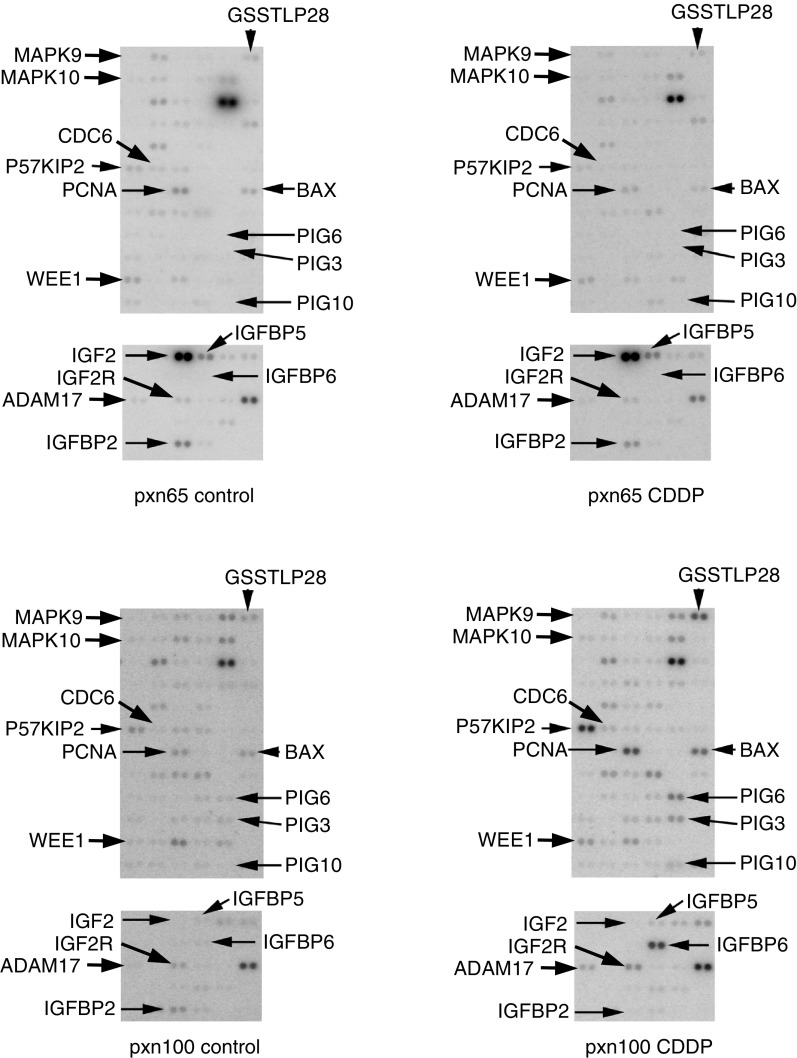
Microarray analysis of RNA samples from pretreatment tumours and 24 h post-CDDP-treatment (6 mg kg^−1^ i.p.). Each gene is spotted in duplicate on the array. Genes increased/decreased >2.5-fold by CDDP-treatment are indicated. For further details see Materials and Methods.

Given the role of p53 in the cell cycle and apoptosis and the observed increase in expression of genes encoding pro-apoptotic proteins (*BAX* and *BAK*), the RNase protection experiments were followed up using a cDNA array of 256 genes, the products of which were specifically associated with cell death or cell cycle regulation. This cDNA array was used to examine the effects on gene expression 24 h after CDDP-treatment (Figure 6

). CDDP treatment of pxn65 tumours decreased the expression of eight genes >2.5-fold. These included two genes the products of which were associated with proliferation (*CDK4* and *PCNA*; Table 1). However, there was no evidence for the increase in expression of any genes following treatment of pxn65 tumours with CDDP. In contrast, CDDP treatment of pxn100 tumours resulted in increased expression of 18 genes and decreased expression of five genes >2.5-fold (Table 1). These changes included increased expression of a number of genes known to be induced following activation of p53 (*PIG3*, *PIG6*, *PIG10*, *PCNA* and *BAX*) that were unchanged in pxn65 cells, an observation consistent with the p53 status of this tumour. In addition, *BAK* expression was increased following CDDP-treatment of pxn100 tumours. The changes in expression of *BAX* and *BAK* detected by microarray were consistent with those observed by RNase protection (Figure 5). We also noted that CDDP-treatment of pxn100 tumours induced the expression by >2.5-fold of genes encoding proteins that regulate the G2M cell cycle checkpoint (*WEE1, CCNB1, CDC25C*) and a gene encoding a protein involved in the anaphase promoting complex in mitosis (*CDC6*). The largest increase in gene expression was the insulin growth factor related gene *IGFBP6* (28-fold) with a smaller increase in another *IGFBP6* related gene *IGF2R* (2.9-fold) and a reduction in the insulin-like growth factor binding protein 2 *(IGFBP2)*. In addition, there was a small decrease in two MAPK-related genes (*MAPK9/JNK9* and *MAPK10/JNK3*).

### Assessment of apoptosis following CDDP treatment

**Figure 7:**
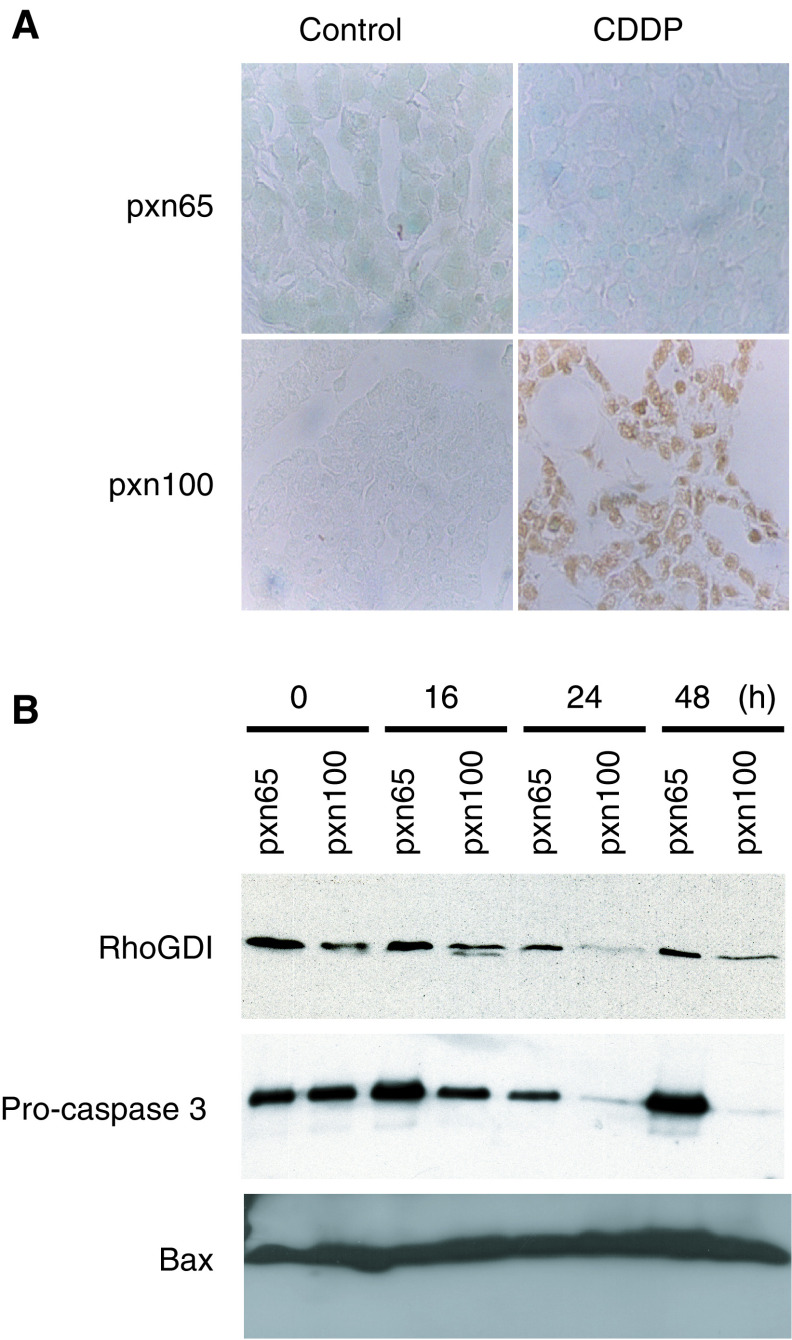
(**A**) *In situ* TUNEL analysis of apoptosis in samples from pretreatment tumours and 24 h post-CDDP treatment (6 mg kg^−1^ i.p.). Nuclei were counterstained with methyl green. A brown nuclear stain indicates a TUNEL positive apoptotic cell. (**B**) Immunoblots (100 *μ*g sample loading) showing BAX, pro-caspase 3 and RhoGDI cleavage in pxn65 and pxn100 tumours 16, 24 and 48 h post-CDDP-treatment.

The single CDDP treatment substantially reduced tumour size and induced *BAX* and *BAK* expression in pxn100 tumour cells (p53 wild type). The products of these genes are pro-apoptotic and are involved in the induction of apoptosis by chemotherapeutic agents (Jones *et al*, 1998; Ionov *et al*, 2000; Zhang *et al*, 2000). Therefore, we examined the induction of apoptosis by measuring apoptosis-associated DNA strand breaks using *in situ* TUNEL analysis between 0 and 96 h post-CDDP-treatment. CDDP-treatment of pxn100 tumours resulted in high levels of TUNEL staining from 16 h onwards (Figure 7A

). In contrast, there was no detectable TUNEL signal at any of the time points following treatment of pxn65 tumours (mutant p53). The positive signal detected in pxn100 tumours was unlikely to be a direct result of cisplatin-induced DNA strand-breaks as they are not recognised by TUNEL unless coupled to an apoptotic response (Chapman *et al*, 1995).

Our observation suggested that CDDP was inducing apoptosis in the pxn100 tumours, but not the pxn65 tumours. To confirm this observation we examined activation of the apoptotic protease cascade by measuring cleavage of pro-caspase 3 and a caspase 3 substrate, Rho-GDI, by Western blotting (Figure 7B). Substantially, decreased levels of pro-caspase 3 were detected 24 and 48 h following treatment in pxn100 tumours only. Similarly, Rho-GDI was also reduced 24 and 48 h after CDDP treatment. These observations were consistent with the TUNEL data and confirmed the significant levels of apoptosis detected in the pxn100 tumours, but not in the pxn65 tumours. In addition, we measured BAX protein expression, but were unable to detect any significant change in BAX protein following CDDP-treatment (Figure 7B).

## DISCUSSION

Our results show the difference in growth delay in response to single doses of CDDP could not be ascribed to differences in DNA damage following treatment as pxn65 had lower levels of detectable DNA adducts than pxn100 tumours following treatment. Pxn100, but not pxn65 tumours, were found to have wild type and functional p53 by sequencing and analysis of p53-regulated gene expression in response to *γ*-irradiation or CDDP-treatment. Increased expression of two pro-apoptotic genes, *BAX* and *BAK*, were detected following treatment of pxn100 tumours, but not pxn65 tumours. These genes have been reported to be involved in the induction of apoptosis following exposure to anticancer agents (Jones *et al*, 1998; Ionov *et al*, 2000; Zhang *et al*, 2000). High levels of apoptosis were detected in pxn100 tumours following CDDP treatment consistent with a p53-dependent apoptotic response as expected from this phenotype. By contrast, in the more sensitive pxn65 tumours, there was no evidence for altered expression of apoptosis regulatory genes or increased apoptosis up to 96 h post-treatment despite the marked response. Therefore, in this *in vivo* model of a curable human ovarian cancer, a robust response to CDDP could be achieved independently of p53 status and did not appear to require the induction of apoptosis.

It has been widely accepted that cell death induced by DNA damage following treatment with anticancer agents is primarily a result of increased apoptosis, and that cells which evade apoptosis will be less responsive to treatment. Although it is evident that defects in apoptosis underpin tumorigenesis and drug resistance, the overall influence of apoptotic defects upon clinical response remains unclear, as it is has not been established that the response of solid tumours in the clinic necessarily involves or requires the induction of apoptosis (Brown and Wouters, 1999; Johnstone *et al*, 2002). A simple paradigm suggests that as long as p53 remains functional, cells damaged by radiation or chemotherapy will either repair or self-destruct. This is largely true in oncogenically transformed rodent cells or lymphoid malignancies where the role of p53 in determining an apoptotic response to radiotherapy or chemotherapy has been clearly demonstrated (Brown and Wouters, 1999). However, the role of p53 in the response of solid tumours to anticancer agents is less clear-cut. To illustrate the complexity, p53^+/+^ mouse embryo fibroblasts immortalised by expression or *E1A* and *RAS* treated with etoposide rapidly die by apoptosis, whereas their p53^−/−^ counterparts do not. However, longer-term clonogenic assays show no difference in overall survival (Brown and Wouters, 1999)*. In vivo* experiments with tumours derived from these isogenic cell lines also demonstrated massive levels of apoptosis and rapidly reduced tumour volume following irradiation of the p53^+/+^ tumours. However, the growth delays for the p53^+/+^ and p53^−/−^ tumours were identical, suggesting that the competence to induce an initial apoptotic response by p53-induction had no effect on the overall response of the tumour (Brown and Wouters, 1999). Similarly, etoposide treatment or irradiation of p53 wild-type HCT116 p21^+/+^ or p21^−/−^ colorectal carcinoma cells demonstrated increased apoptosis in the p21^−/−^ line, but no difference in clonogenic survival (Wouters *et al*, 1997). Expression of the anti-apoptotic *BCL-2* gene in the p21^−/−^ cell line reduced the level of apoptosis compared to the parental HCT116 p21^−/−^ cells following irradiation; however, again there was no difference in clonogenic survival (Wouters *et al*, 1999). *In vivo* experiments also illustrated this effect, with p53 wild-type HCT116 p21^−/−^ cells exhibiting high levels of apoptosis (90% of the tumour by TUNEL assay) following irradiation. Expression of BCL-2 eliminated the apoptotic response, but did not influence the overall response of the tumour as the growth delay was similar for both tumours (Wouters *et al*, 1999).

In some respects our observations are comparable to the *in vivo* study of Wouters *et al* (1999). The pxn100 p53 wild-type germ cell ovarian tumour that might be expected to be CDDP sensitive exhibited the ‘classical’ response of p53 induction, altered p53-dependent gene expression and high levels of apoptosis that was followed by a 50% reduction in tumour volume. We also noted that CDDP induced the expression of genes encoding proteins that regulate the G2/M cell cycle checkpoint. Induction of a cell cycle arrest and a specific cell cycle check point at the boundary of G2 and M phases has been implicated in apoptosis induction (Eastman, 1999). If the cellular damage induced in G1/S and G2/M, such as a DNA adduct, is detected but irreparable, cells would be eliminated via apoptosis to prevent them entering mitosis. This suggests that following treatment of pxn100 cells, DNA damage is detected and the cells respond through induction of p53. Irreparably damaged cells are rapidly eliminated by an apoptotic mechanism and in the absence of further treatment the remaining cells survive and the tumour regrows. Subsequent treatments would prevent the regrowth and cause further cell death (apoptosis), as is the case with pxn100 xenografts where additional CDDP treatment on days 7, 14 and 28 results in cure (Harrap *et al*, 1990). A study examining gene expression by cDNA microarray in a pair of p53^+/+^ ovarian adenocarcinoma cell lines (KFr and its acquired CDDP resistant counterpart KFrP200) showed a number of gene changes (Sakamoto *et al*, 2001). In particular, CDDP resistance was associated with elevated *GST-pi* expression and increased expression of several insulin growth factor receptor genes. Sensitivity was associated with expression of certain cell cycle and apoptosis genes, including *BAK* and *CDC25C* (Sakamoto *et al*, 2001) both of which were upregulated in pxn100 following CDDP exposure. CDDP treatment of pxn100 also induced several members of the insulin growth factor gene family, which have been implicated in the growth and development of ovarian cancer (Sawiris *et al*, 2002). Additionally, the expression of two MAPK-related JNK genes were decreased by CDDP treatment and this pathway may be involved in sensing platinum-dependent DNA damage (Potapova *et al*, 1997).

Paradoxically, the epithelial pxn65 tumour with a mutant p53 that might, on the basis of some studies, be predicted to be far less sensitive to CDDP was actually even more sensitive to CDDP than pxn100 and responded in the absence of functional p53 or detectable apoptosis. There were few significant or meaningful changes in genes encoding regulators of the cell cycle or apoptotic cell death following CDDP treatment in this tumour. Decreased tumour size was detected later than that observed for pxn100. One explanation is that these tumours failed to recognise DNA damage resulting from CDDP treatment or that the loss of p53 resulted in the tumour cells being unable to respond to the damage. The absence of p53-dependent cell cycle checkpoint regulation following treatment would allow the tumours to acquire chromosomal damage until the damage was sufficient to result in a mitotic catastrophe, that is, death as a consequence of mechanical damage (necrosis) rather than induction of a rapid self-destruct signal (apoptosis). This would also explain the slow decrease in tumour volume and the widely spaced dosing of CDDP on days 0, 49 and 77 that were optimal for curing pxn65 tumours (Harrap *et al*, 1990, unpublished). Recently, it has been demonstrated that certain types of DNA damage, such as alkylation, can stimulate extensive necrotic cell death *in vitro* without a requirement for functional p53, BAX/BAK or caspase activation (Zong *et al*, 2004). The absence of a requirement for p53 would also be consistent with clinical studies where p53 status appears to have at best a marginal effect on response and survival to platinum-based treatment of ovarian tumours (Lavarino *et al*, 2000; Reles *et al*, 2001). Interestingly, treatment of ovarian cancer patients by reintroduction of functional p53 into the ovarian cancer cells using an adenoviral vector, did not improve clinical response to treatment with carboplatin and paclitaxel, despite preclinical studies showing promise. One explanation proffered was that expression of a functional p53 restores the ability to recognise, repair and respond to treatment-induced DNA damage and effectively attenuates or neutralises the effects of concomitantly administered drugs (Zeimet and Marth, 2003).

In conclusion, previous studies of established cell lines have demonstrated that apoptotic response and overall treatment sensitivity can be distinct from each other. These observations refute the paradigm that anticancer agents kill cells predominantly through apoptosis and cells resistant to apoptosis will be less responsive to treatment. For the first time we demonstrate, using two *in vivo* models of curable ovarian cancer that had not been previously adapted to tissue culture, that a marked response to a clinically relevant cytotoxic anticancer agent could be achieved in the presence, and perhaps more importantly, in the absence of functional p53 where apoptosis was undetectable. Further investigation of the role of cell cycle regulation and DNA repair, together with more detailed and larger microarray studies are warranted in these two tumour models, which may be valuable for the exploration of *in vivo* responses.
